# Impact of Glucagon-like Peptide-1 Receptor Agonists on Mental Illness: Evidence from a Mendelian Randomization Study

**DOI:** 10.3390/ijms26062741

**Published:** 2025-03-18

**Authors:** Longgang Xiang, Ying Peng

**Affiliations:** State Key Laboratory of Bioactive Substances and Functions of Natural Medicines, Institute of Materia Medica, Chinese Academy of Medical Sciences & Peking Union Medical College, Beijing 100050, China; xianglonggang@imm.ac.cn

**Keywords:** GLP1R agonists, drug target, mental illness, Mendelian randomization, genome-wide association studies

## Abstract

Emerging evidence suggests that glucagon-like peptide-1 receptor (GLP1R) agonists may have potential benefits for mental illnesses. However, their exact effects remain unclear. This study investigated the causal relationship between glucagon-like peptide-1 receptor agonist (GLP1RA) and the risk of 10 common mental illnesses, including attention deficit and hyperactivity disorder, anorexia nervosa, anxiety disorder, autism spectrum disorder, bipolar disorder, major depressive disorder, post-traumatic stress disorder, schizophrenia, cannabis use disorder, and alcohol use disorder. We selected GLP1RA as the exposure and conducted a Mendelian randomization (MR) analysis. The cis-eQTLs of the drug target gene *GLP1R*, provided by eQTLGen, were used to simulate the pharmacological effects of GLP1RA. Type 2 diabetes and BMI were included as positive controls. Using data from both the Psychiatric Genomic Consortium and FinnGen, we conducted separate MR analyses for the same disease across these two independent databases. Meta-analysis was used to pool the results. We found genetic evidence suggesting a causal relationship between GLP1RA and a reduced risk of schizophrenia [OR (95% CI) = 0.84 (0.71–0.98), *I*^2^ = 0.0%, common effects model]. Further mediation analysis indicated that this effect might be unrelated to improvements in glycemic control but rather mediated by BMI. However, the findings of this study provide insufficient evidence to support a causal relationship between GLP1RA and other mental illnesses. Sensitivity analyses did not reveal any potential bias due to horizontal pleiotropy or heterogeneity in the above results (*p* > 0.05). This study suggests that genetically proxied activation of glucagon-like peptide-1 receptor is associated with a lower risk of schizophrenia. GLP1R is implicated in schizophrenia pathogenesis, and its agonists may exert potential benefits through weight management. Our study provides useful information for understanding the neuropsychiatric effects of GLP1RA, which may contribute to refining future research designs and guiding clinical management. Moreover, our findings could have significant implications for overweight individuals at high risk of schizophrenia when selecting weight-loss medications. Future research should further investigate the potential mechanisms underlying the relationship between GLP1RA and schizophrenia.

## 1. Introduction

Glucagon-like peptide-1 (GLP-1) is an incretin hormone that exerts various physiological functions throughout the body, most notably in regulating blood glucose levels and controlling appetite [1]. Glucagon-like peptide-1 receptor agonist (GLP1RA) helps to regulate blood glucose levels by mimicking endogenous GLP-1 [2]. Semaglutide, originally developed for the treatment of diabetes, was approved by the FDA on 4 June 2021 as a weight-loss medication due to its ability to activate glucagon-like peptide-1 receptor (GLP1R) in the central nervous system [3], reducing food intake and promoting weight loss [4]. With advancements in research, GLP-1 has demonstrated promising potential in areas like cardioprotection and neuroprotection [5], generating significant interest in its effects beyond lowering of glucose and weight reduction.

Mental illnesses refer to a group of severe disorders that lead to brain dysfunction, causing significant public health concerns worldwide [6]. Affecting approximately 18% of the global population [7], they contribute to substantial mortality and economic burden [7,8]. Affected individuals experience disturbances in mood, cognition, or behavior. Mood improvement was initially observed in diabetic patients treated with GLP1RA. A large-scale cohort study of 10,690 diabetes patients over 6 to 7 years found that GLP1RA users had a lower incidence of depressive and anxiety disorders compared to non-users [9]. This observation sparked interest in the potential use of GLP1RAs for managing mental illnesses. GLP1Rs are widely distributed in the brain and play a role in regulating neuronal function [5,10,11], synaptic plasticity [12], and neurotransmitter release [13]. Although evidence from preclinical studies and clinical trials remains limited, liraglutide has been shown to improve executive function and memory in patients with major depressive disorder and bipolar disorder [14] as well as memory in individuals with prediabetes or early-stage type 2 diabetes and obesity [15]. In animal models, GLP1RAs have been found to enhance neurogenesis and regulate stress response circuits associated with mood disorders [16]. In addition, GLP1RAs have also demonstrated anti-inflammatory and antioxidant properties [13], which may offer potential benefits for a variety of mental illnesses. Overall, emerging evidence suggests that GLP1RA may have neuropsychiatric benefits. However, there is currently no direct evidence confirming the neuropsychiatric effects of GLP1RA in humans, and high-quality randomized controlled trials (RCTs) are still lacking. Most existing findings come from observational studies, and further research is still in its early stages. Recently, an observational study with a cohort of up to 2.4 million participants systematically examined the association between GLP1RAs and 175 health outcomes [17]. This study stands as the most extensive investigation of GLP1RAs to date. We took particular interest in the section on mental illnesses and found that the study reported that GLP1RA may reduce the risk of alcohol use disorder (AUD), cannabis use disorder (CUD), schizophrenia (SZ), and other mental health outcomes. Establishing a causal link between GLP1RAs and mental illness management could pave the way for new clinical indications. Equally important is the need to assess the potential psychiatric risks associated with long-term GLP1RA use. Therefore, further exploration of the causal relationships and underlying mechanisms between GLP1RAs and psychiatric disorders is warranted.

Mendelian randomization (MR) is a genetic study that assesses causal relationships between exposure and outcome [18]. Observational studies are often subject to biases such as confounding and reverse causation. In contrast, MR prevents these biases by utilizing the random allocation of genetic variants from parents to offspring during conception, thereby offering robust causal inference capabilities [19]. Based on rigorous instrumental variable selection procedure, drug target MR uses genetic variants to simulate the agonistic effects of drug targets, helping to evaluate their long-term exposure impact on diseases [20,21]. By selecting outcomes of interest, it can be used to study the potential efficacy and safety of new drug targets [22,23] as well as to explore the repurposing potential and adverse effects of existing drugs [20]. When RCTs are unavailable, drug target MR can provide high-level human genetic evidence to support drug repositioning and the identification of adverse effects [24]. We have observed that several MR studies have explored the associations between GLP1RA and conditions such as stroke, Alzheimer’s disease, osteoarthritis, and cancer [25,26,27,28], contributing to a comprehensive evaluation of GLP1RA. Drug target MR can provide valuable insights into the clinical management of GLP1RA in relation to mental illnesses.

Overall, accumulating evidence suggests an association between GLP1RA and the risk of various mental illnesses, though the exact causal relationship remains unknown. In this context, we hypothesize that GLP1RA use may influence the risk of mental illness. This study aims to investigate the causal relationships between GLP1RA and 10 common mental illnesses: AUD, CUD, SZ, attention deficit and hyperactivity disorder (ADHD), anorexia nervosa (AN), anxiety disorder (ANX), autism spectrum disorder (ASD), bipolar disorder (BD), major depressive disorder (MDD) and post-traumatic stress disorder (PTSD). Specifically, we first perform drug-target MR analyses to examine the causal relationship between GLP1RA use and the aforementioned disorders. Additionally, for the identified causal relationships, we adopt MR mediation analysis to explore the potential mediating roles of glycemic control and weight management, which aids in understanding the underlying biological mechanisms of GLP1RA. This is the first systematic MR study investigating GLP1RA and the risk of mental illnesses. This research not only contributes to exploring the potential indications of GLP1RA but also provides valuable insights for assessing the risks of mental illnesses with their use.

## 2. Results

### 2.1. Selection of Genetic Instruments

**Figure 1:**
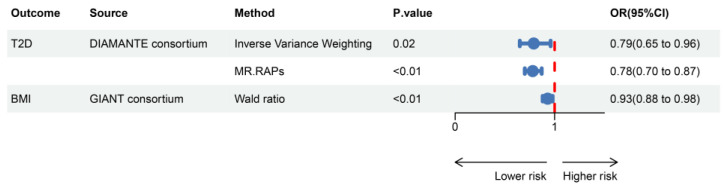
Positive control analysis showing estimates of causal effects between GLP1RA and BMI/T2D. T2D, type 2 diabetes; MR.RAPs, Mendelian randomization robust adjusted profile scores; OR, odds ratio; CI, confidence interval.

The instrumental variables used to simulate the pharmacological effects of GLP1R activated in this study all have F-statistics greater than 40, indicating no bias caused by weak instruments. In the positive control analysis, GLP1RA significantly reduces the risk of T2D [OR (95% CI) = 0.79 (0.65–0.96), *p* = 0.02] and demonstrates an effect in lowering BMI [OR (95% CI) = 0.93 (0.88–0.98), *p* < 0.01]. These results further validate the effectiveness of the selected instrumental variables (Figure 1).

### 2.2. Causal Relationship Between GLP1RA and Mental Illnesses

**Figure 2:**
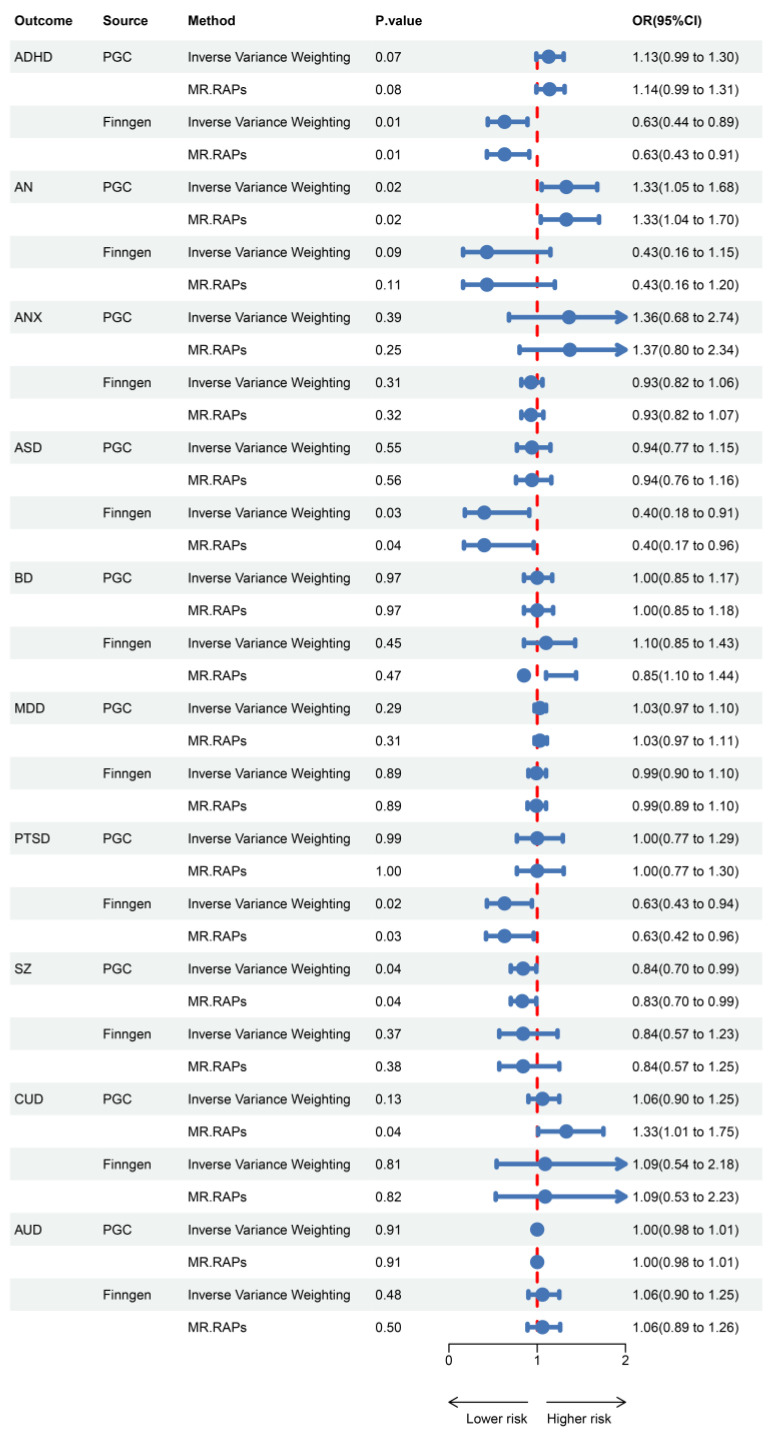
Associations of genetically predicted GLP1RA with mental illnesses.

Results for the causal effects of GLP1RA on mental illnesses are shown in Figure 2. In the MR analysis with outcomes data provided by the Psychiatric Genomics Consortium (PGC), the primary method indicated that GLP1RA was associated with an increased risk of AN [OR (95% CI) = 1.33 (1.05–1.68), *p* = 0.02] and a decreased risk of SZ [OR (95% CI) = 0.84 (0.70–0.99), *p* = 0.04]. In the FinnGen dataset, GLP1RA was associated with a decreased risk of ADHD [OR (95% CI) = 0.63 (0.44–0.89), *p* = 0.01], ASD [OR (95% CI) = 0.40 (0.18–0.91), *p* = 0.03], and PTSD [OR (95% CI) = 0.63 (0.43–0.94), *p* = 0.02]. MR-Egger, weighted median, weighted mode, and simple mode methods, which are based on different statistical assumptions, provided consistent estimates of effect direction (Supplementary Tables S1 and S2). This consistency enhances the reliability of the causal inference. There was no evidence of heterogeneity or horizontal pleiotropy (Supplementary Tables S3 and S4), indicating that our analysis was robust.

### 2.3. Meta-Analysis

**Figure 3:**
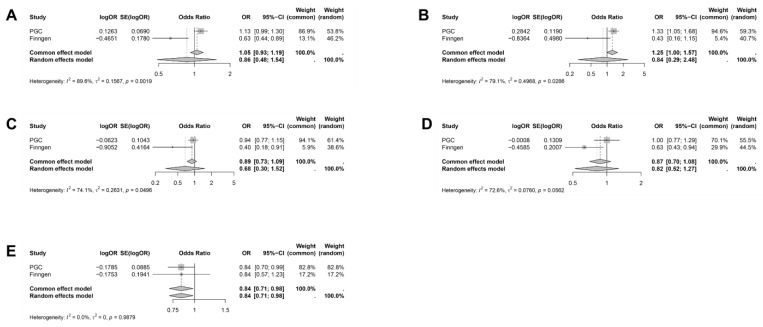
Meta-analysis of the causal effect of GLP1RA on mental illnesses. (**A**) ADHD, (**B**) AN, (**C**) ASD, (**D**) PTSD, (**E**) SZ. The causal effect estimates were derived from the inverse variance weighting (IVW) method.

We then focused on the outcomes that demonstrated at least one statistically significant association. Specifically, we performed a meta-analysis pooling MR results conducted separately in PGC and FinnGen. This helped to increase statistical power and the breadth of analysis while minimizing potential bias (Figure 3).

A causal relationship between GLP1RA and reduced SZ risk was found [OR (95% CI) = 0.84 (0.71–0.98), *I*^2^ = 0.0%, Common effects model]. This is considered as the primary finding of our study. However, there is insufficient evidence to support a causal relationship between GLP1RA and the risk of ADHD [OR (95% CI) = 0.86 (0.48–1.54), *I*^2^ = 89.6%, Random effects model], AN [OR (95% CI) = 0.84 (0.29–2.48), *I*^2^ = 79.1%, Random effects model], ASD [OR (95% CI) = 0.68 (0.30–1.52), *I*^2^ = 74.1%, Random effects model], and PTSD [OR (95% CI) = 0.82 (0.52–1.27), *I*^2^ = 72.6%, Random effects model].

### 2.4. Meditation Analysis

**Figure 4:**
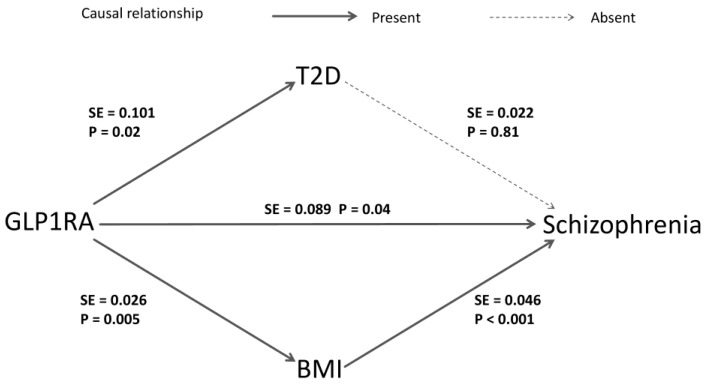
The mediation analysis of GLP1RA on SZ risks. The arrows indicate the causal direction from cause to effect, with solid lines representing the presence of a causal relationship and dashed lines indicating the absence of one. The *p* and SE values are derived from the estimates of the IVW method in the corresponding MR analysis. The delta method uses the SE values to generate the standard error in the coefficient product test.

After identifying the causal relationship, conducting MR mediation analysis could help to discover underlying biological mechanisms. We primarily considered the potential mediating roles of T2D and BMI (Figure 4). We found no causal relationship between T2D and the risk of SZ [OR (95% CI) = 0.99 (0.95–1.04), *p* = 0.81], while a causal relationship between BMI and SZ was found [OR (95% CI) = 0.82 (0.75–0.89), *p* < 0.001]. In the framework of the two-step MR method, this indicates that the effect of GLP1RA in reducing SZ risk is unlikely to be mediated by glycemic control but may be related to BMI. The coefficient product test using the delta method further supports the mediation effect of BMI (*p* < 0.05). Detailed results are provided in Supplementary Tables S5 and S6.

## 3. Discussion

The intriguing link between neuroscience and endocrinology holds the potential to lead to groundbreaking therapies for mental illnesses. In recent years, there has been an increasing interest in the effects of GLP1RA beyond their role in regulating blood glucose. Although observed improvements in mood among diabetic patients receiving GLP1RA therapy have offered valuable insights, the ultimate effects and underlying mechanisms remain unclear. In this study, we performed a robust Mendelian randomization analysis and systematically investigated for the first time the causal relationship between GLP1RA and the risk of 10 mental illnesses. We found that genetically proxied GLP1R activation was associated with a reduced risk of schizophrenia and further mediation analysis suggests that this effect might not be related to glucose control but mediated by BMI.

Cognitive impairment is a core feature of schizophrenia [29], with current antipsychotic therapies primarily addressing positive symptoms, while cognitive deficits remain largely unaddressed [30]. Currently, the clinical application of GLP1RAs in schizophrenia management has primarily focused on their role in weight regulation. When co-administered with antipsychotic medications such as lithium, clozapine, and olanzapine, GLP1RAs have demonstrated the ability to mitigate metabolic disturbances and prevent cognitive deterioration [31]. However, research exploring the cognitive-enhancing effects of GLP1RA remains scarce and is predominantly confined to preclinical studies. Preclinical evidence has shown that GLP1RA can substantially improve spatial learning and memory [32,33,34,35,36,37,38]. In murine models, liraglutide has been observed to prevent MK-801-induced schizophrenia-like behaviors [39]. These findings support the potential benefits of GLP1RA for schizophrenia. Notably, GLP1RA has also been shown to enhance cognitive function in models of diabetes and neurodegenerative diseases, such as Alzheimer’s disease [40]. This suggests that GLP1RA may have a direct effect on cognitive function independent of glucose normalization. In summary, these results provide compelling evidence that targeting GLP1R may have a positive impact on the clinical management of schizophrenia. This finding is consistent with the conclusions of large-scale observational studies [17]. This MR study suggests that the use of GLP1RA is associated with a reduced risk of schizophrenia, and the evidence from the animal models supports the potential of GLP1R as a therapeutic target for schizophrenia. Despite the genetic evidence, further studies are needed to ultimately confirm these findings and investigate the underlying molecular mechanisms.

Mediation analysis suggests that the mechanism by which GLP1RA reduces the risk of schizophrenia is related to weight management. Some genome-wide cross-trait analysis uncovers a shared genetic architecture between schizophrenia and BMI, indicating a complex biological connection between them [41,42,43]. While the molecular pathways of GLP1RA signaling have been identified, the direct mechanisms by which they enhance cognitive processes remain unclear. Current research suggests that these mechanisms may involve biological processes such as promoting neurogenesis and synaptic plasticity, reducing neuroinflammation, enhancing insulin signaling, preventing neuronal apoptosis, and minimizing oxidative stress [44]. The mechanisms by which GLP1RA exerts potential benefits on schizophrenia through weight management remain unclear, but clues can be found in the pathophysiology of schizophrenia. Weight loss is commonly associated with reduced systemic inflammation, and neuroinflammation is considered a key factor in the pathogenesis of schizophrenia. One possible mechanism is that GLP1RA may reduce inflammation through weight loss, thereby offering protective effects on the nervous system. This hypothesis is plausible. In current clinical practice, obesity has been associated with adverse psychiatric outcomes in individuals with SZ [45,46]. It is important to note that current studies on the cognitive-improving effects of GLP1RA are mainly based on diabetes and Alzheimer’s disease models. Future explorations of the mechanisms should focus more on schizophrenia models to better understand the potential therapeutic effects of GLP1RA.

Another contribution of this study is the lack of evidence for a causal relationship between GLP1RA and the risk of other mental disorders. GLP1RAs are demonstrating significant development potential, and clinical evidence reporting the long-term usage risks for mental disorders remains relatively scarce. It is important to evaluate them as comprehensively as possible. In our study, there was insufficient evidence to support a causal relationship between GLP1RA use and other mental illnesses. Although in the analysis with outcomes from PGC, GLP1RA use was associated with an increased risk of AN, and in the analysis with outcomes from FinnGen, GLP1RA use was associated with a reduced risk of ADHD, ASD, and PTSD, the meta-analysis failed to reach statistical significance. These findings will be regarded as “suggestive evidence” and may provide promising directions for future in-depth studies. We noted that larger research interests are primarily focused on anxiety, depression, eating disorders, and addictive disorders. Improvement in anxiety and depression is of particular interest. Preclinical studies have provided preliminary evidence for the antidepressant and anxiolytic properties of GLP1RA [47], and several clinical studies have reported improvements in depressive symptoms following GLP1RA treatment [48,49]. However, there is also evidence reporting the emergence of anxiety and depressive symptoms associated with the use of semaglutide [50,51]. There is even evidence from the FDA Adverse Event Reporting System (FAERS) suggesting that the use of semaglutide may be associated with an increased risk of suicidal ideation [52]. This makes the relationship between GLP1RA use and anxiety and depression unclear. Additionally, our study found suggestive evidence indicating an increased risk of anorexia nervosa. In contrast, liraglutide is considered a safe and effective treatment for bulimia nervosa and binge eating disorder [53]. These findings suggest the need for continued attention to the effects of GLP1RA use in the management of eating disorders. Another promising research direction for GLP1RA is their potential in controlling the risk of addiction disorders. An observational study involving 2.4 million people supports this [17], but as they pointed out in their discussion, “observational studies cannot establish causality”. Currently, there is not enough investigation to determine the exact effects of long-term use. In fact, the conclusions of the vast majority of current studies on the above diseases come from observational research, which cannot establish causality. Research on the psychiatric benefits of GLP1RAs is still in its early stages. Therefore, as a result of a MR study, we recommend conducting more large-scale, long-term follow-up, multi-center randomized controlled trials in the future to validate all of our conclusions. This will contribute to drug repositioning of GLP1RAs and the prediction of adverse reactions, ultimately translating into clinical practice. After confirming the causal relationship, further investigation into the mechanisms is needed to distinguish whether the neuropsychiatric effects of GLP1RAs are direct or mediated through the improvement of the original indications (T2D and obesity). This distinction may be important, as it may reveal a unique therapeutic mechanism of GLP1RAs for mental illnesses.

Our study has several strengths. First, to our knowledge, this is the first study to apply drug target MR to systematically investigate the effects of GLP1RA on mental illnesses, offering stronger causal inference compared to traditional observational studies. Second, due to the differences in GLP1R expression between mice and humans, it is challenging to extend preclinical research findings to clinical trials [54]. Our analysis, based on large-scale human GWAS summary-level data, can provide more valuable insights beneficial to clinical practice. Finally, triangulation across multiple databases and the design of positive controls further improved the design of drug target MR. And meta-analysis helps to avoid potential biases.

However, it must be noted that, like other MR studies on drug targets, our conclusions are subject to several limitations. First, unlike drug administration in clinical practice, which typically occurs over a shorter timeframe, genetic variants used as proxies for drug effects represent lifelong cumulative effects of small-scale perturbations to the drug target [55]. This means that the odds ratios presented in this study cannot be directly used to guide clinical practice. In fact, estimates from genetic proxy analyses are generally smaller than those observed in clinical practice [56]. Furthermore, when larger scale tissue-specific eQTL data become available, it will be important to explore the relationship between GLP1RA and the risk of mental illness across more tissues for a more in-depth evaluation. The study population was limited to individuals of European ancestry, so caution is needed when generalizing the findings to other populations.

## 4. Materials and Methods

### 4.1. Study Design

**Figure 5:**
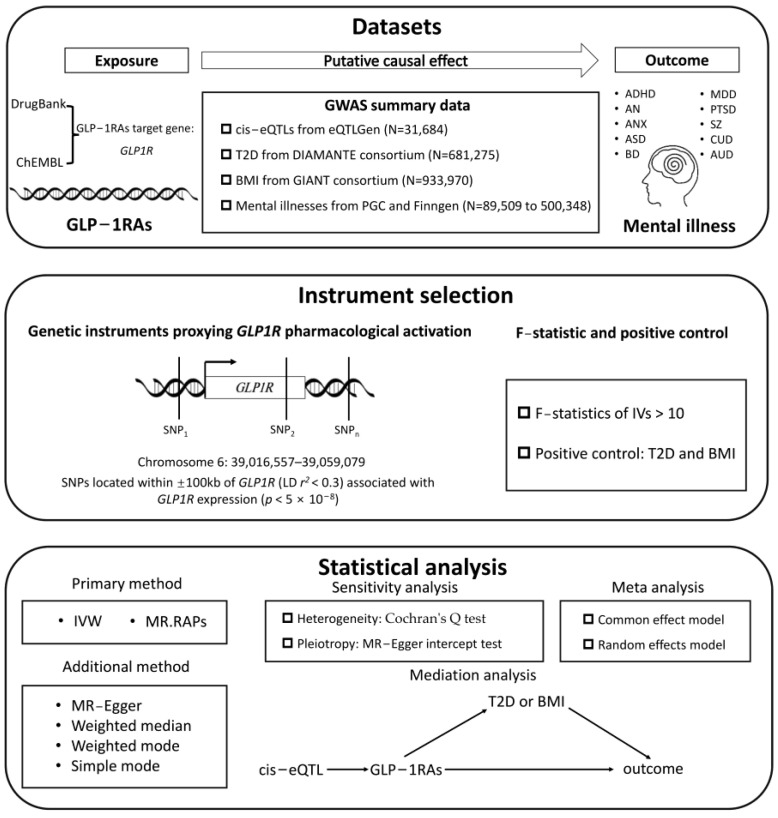
The overall study design flowchart.

In this study, we implemented the following workflow: First, we obtained the drug target gene *GLP1R* for GLP1RA from DrugBank and ChEMBL, and we then used cis-eQTLs in blood to proxy the pharmacological effects of the target activation. A series of stringent criteria for instrumental variable selection were established. Subsequently, two sample MR analyses were conducted using psychiatric disorder datasets from two independent databases to explore potential causal relationships. Meta-analysis was employed to pool the results and draw final conclusions. Finally, mediation analyses were performed to investigate whether the discovered causal relationships were mediated by glycemic improvement or weight management. The overall study design is illustrated in Figure 5.

### 4.2. Data Source

Exposure and outcome data were derived from publicly available GWAS studies. To minimize potential bias caused by population diversity, the analysis was restricted to individuals of European ancestry. Table 1 provides further information on the genetic data resources. Further details on the disease endpoints are available in the corresponding publications. Ethics approvals were obtained by the original studies from relevant authorities, and informed consent was provided by all participants.

### 4.3. Genetic Instrumental Selection

Information regarding the pharmacologically active protein targets and corresponding encoding genes was retrieved from the DrugBank and the ChEMBL databases [71,72]. In this study, GLP1RA was selected as the exposure. Instrumental variables representing the expression of the drug target gene in blood was obtained from cis-eQTLs provided by eQTLGen. Specifically, SNPs significantly associated (*p* < 5 × 10^−8^) with the target gene *GLP1R* (chromosome 6, base position: 39,016,557–39,059,079) within a 100 kb upstream and downstream window were selected. Subsequently, clumping was performed with a linkage disequilibrium (LD) threshold of *r*^2^ < 0.3 [73,74]. Instrumental strength was ensured when the F-statistic exceeded 10, indicating a robust association with the exposure traits [75].

The decades-long use of GLP1RAs, principally acylated peptides such as liraglutide and semaglutide, for the treatment of obesity and T2D [76] has demonstrated their efficacy and safety. Accordingly, we designed a positive control MR analysis, where their expected effects on the outcomes can validate the effectiveness of the selected instrumental variables [77]. It is worth noting that the instrumental variables could not be directly identified in the summary GWAS data for BMI as the outcome in the MR analysis. To address this issue, we searched for proxies within a 100 kb window around the variants using an LD threshold of *r*^2^ > 0.85 to identify proxy SNPs for the analysis [74]. The setting of a positive control ensured the validity of the instrumental variables employed in this study.

### 4.4. MR and Sensitivity Analysis

We performed a two-sample MR analysis to estimate the association between GLP1RA and mental illnesses. In this study, inverse variance weighting (IVW) and Mendelian Randomization Robust Adjusted Profile Scores (MR.RAPs) were selected as the primary methods. The IVW method assumes balanced pleiotropy and applies a multiplicative random effects model to provide consistent estimates of causal effects [78]. In contrast, the MR.RAPs method utilizes robust adjusted profile scores for statistical inference, though it may still be biased due to unbalanced pleiotropy [79]. When the number of SNPs is less than 2, the Wald ratio is a feasible method. In addition, weighted mode [79], MR-Egger [80], weighted median [81], and simple mode were used as complementary methods.

We initially used mental illnesses data from PGC as the outcome and subsequently replicated the analysis using the summary-level GWAS data from the Finngen Consortium (R12) (https://www.finngen.fi/en). The FinnGen study is an extensive genomics initiative that has analyzed over 500,000 samples from the Finnish biobank, examining the correlation between genetic variation and health data to gain insights into disease mechanisms and predispositions [61]. This project involves Finnish research groups, biobanks, and global industry partners. In addition, all MR analyses were performed with sensitivity tests. We used Cochran’s Q test [82] to assess heterogeneity and MR-Egger intercept [83] to detect horizontal pleiotropy.

### 4.5. Meta Analysis

Diseases that showed statistical significance in at least one of the two independent analyses for the same mental illness were further pooled using meta-analysis, which will help integrate causal conclusions across different databases. The results from the meta-analysis will serve as the primary basis for reporting the causal relationship between GLP1RA and specific mental illness outcomes in this study.

### 4.6. Mediation Analysis

Although the meta-analysis ultimately identified a causal relationship between GLP1RA and mental illness, the underlying mechanism remains unclear. It is necessary to consider whether the discovered causal relationship is related to the effects of GLP1RA on improving glucose levels or reducing weight; if not, it may suggest a direct effect of GLP1RA on the risk of mental illnesses.

To explore this, we conducted a preliminary investigation using multivariable Mendelian randomization [84], incorporating both the two-step MR method and the coefficient product test. The two-step method requires the existence of causal relationships between the exposure, mediator, and outcome in pairs, otherwise, the mediator may not exhibit a true mediation effect [85]. In the positive control, we identified a causal relationship between GLP1RA and both T2D and BMI. The causal relationship between the potential mediator and the target mental illness will be further considered. The selection of instrumental variables and the MR analysis procedure remain as described previously. If there is no causal relationship between the potential mediator and the outcome, it is unlikely that the causal effect from exposure to outcome is mediated by this potential mediator. If identifies causal relationship, we then apply the coefficient product test to further examine the mediation effect [86].

### 4.7. Statistical Analysis

Within our MR analysis, the results were quantified as odds ratios (ORs) with 95% confidence intervals (CIs), where the OR represents the change in susceptibility to mental illnesses per standard deviation increase in genetically predicted GLP1R expression. Given that IVW and MR.RAPs were the primary estimation models in this study, results were considered statistically significant when both methods reported *p* < 0.05 with consistent effect direction estimates across multiple analytical approaches. Additionally, conclusions were regarded as robust when no heterogeneity or pleiotropy was found (*p* > 0.05).

For the meta-analysis, we used *I*^2^ as an indicator of heterogeneity between incorporated studies. When it was below 50%, a common effect model was applied; otherwise, a random effects model was employed, which helped address heterogeneity in data analysis. If the OR obtained from the meta-analysis was consistently less than 1 or greater than 1, the exposure was considered associated with a reduction or increase in disease risk, which constitutes the main finding of this study.

The two-step MR method involves conducting MR analyses between exposure, outcome, and potential mediators in pairs. For each independent MR analysis, the statistical analysis methods are consistent with the standards previously described. The standard error used in the coefficient product test is generated by the delta method and statistical significance was determined by the *p* obtained from this test, with *p* < 0.05 indicating the presence of a mediation effect. All analyses were based on the “TwoSampleMR” R package (version 0.5.8).

## 5. Conclusions

This MR study suggests that GLP1RAs are causally associated with a reduced risk of schizophrenia, providing genetic evidence for their use in the clinical management of schizophrenia. This neuropsychiatric benefit may be associated with weight management effects. Our findings may also provide valuable guidance for selecting weight-loss medications for overweight individuals at high risk of schizophrenia.

## Figures and Tables

**Table 1:** Data source used in the current study.

Phenotypes	Source	Sample Size	Reference
eQTL for *GLP1R*	eQTLGen consortium	31,684	[57]
T2D	DIAMANTE consortium ^1^	681,275	[58]
BMI	GIANT consortium ^2^	933,970	[59]
ADHD	PGC ^3^	38,691/225,534	[60]
	Finngen	4452/490,708	[61]
AN	PGC	16,992/72,517	[62]
	Finngen	546/499,802	[61]
ANX	PGC	7016/21,761	[63]
	Finngen	35,875/444,414	[61]
ASD	PGC	18,381/46,350	[64]
	Finngen	888/362,304	[61]
BD	PGC	41,917/413,466	[65]
	Finngen	8946/434,831	[61]
MDD	PGC	43,204/138,884	[66]
	Finngen	59,333/434,831	[61]
PTSD	PGC	32,428/206,655	[67]
	Finngen	3444/444,414	[61]
SZ	PGC	30,989/113,075	[68]
	Finngen	7234/484,776	[61]
CUD	PGC	20,916/363,116	[69]
	Finngen	1168/491,508	[61]
AUD	PGC	141,932	[70]
	Finngen	20,597/479,751	[61]

^1^ DIAMANTE consortium, Diabetes Meta-Analysis of Trans-Ethnic Association Studies consortium; ^2^ GIANT consortium, Genetic Investigation of ANthropometric Traits consortium; ^3^ PGC, Psychiatric Genomics Consortium. And other explanations of the abbreviations could be found in the manuscript.

## Data Availability

All datasets produced in this study are included in the manuscript and its Supplementary Materials.

## Supplementary Materials

The supporting information can be downloaded at: https://www.mdpi.com/article/10.3390/ijms26062741/s1.

## Abbreviations

The following abbreviations are used in this manuscript:

GLP-1	Glucagon-like peptide-1
GLP1R	Glucagon-like peptide-1 receptor
GLP1RA	Glucagon-like peptide-1 receptor agonist
MR	Mendelian randomization
OR	Odds ratio
CI	Confidence interval
LD	Linkage disequilibrium
RCT	Randomized controlled trial
T2D	Type 2 diabetes
ADHD	Attention deficit hyperactivity disorder
AN	Anorexia nervosa
ANX	Anxiety
ASD	Autism spectrum disorder
BD	Bipolar disorder
MDD	Major depressive disorder
PTSD	Post-traumatic stress disorder
SZ	Schizophrenia
CUD	Cannabis use disorder
AUD	Alcohol use disorder
IVW	Inverse variance weighting
MR.RAPs	Mendelian randomization robust adjusted profile scores

## References

[1] Calsolaro V., Edison P. (2015). Novel GLP-1 (Glucagon-Like Peptide-1) Analogues and Insulin in the Treatment for Alzheimer’s Disease and Other Neurodegenerative Diseases. CNS Drugs.

[2] Müller T.D., Finan B., Bloom S.R., D’Alessio D., Drucker D.J., Flatt P.R., Fritsche A., Gribble F., Grill H.J., Habener J.F. (2019). Glucagon-like peptide 1 (GLP-1). Mol. Metab..

[3] Wilding J.H., Batterham R.L., Calanna S., Davies M., Van Gaal L.F., Lingvay I., McGowan B.M., Rosenstock J., Tran M.D., Wadden T.A. (2021). Once-Weekly Semaglutide in Adults with Overweight or Obesity. N. Engl. J. Med..

[4] Aggarwal R., Vaduganathan M., Chiu N., Bhatt D.L. (2021). Potential implications of the FDA approval of semaglutide for overweight and obese adults in the United States. Prog. Cardiovasc. Dis..

[5] Andersen A., Lund A., Knop F.K., Vilsbøll T. (2018). Glucagon-like peptide 1 in health and disease. Nat. Rev. Endocrinol..

[6] Charlson F., van Ommeren M., Flaxman A., Cornett J., Whiteford H., Saxena S. (2019). New WHO prevalence estimates of mental disorders in conflict settings: A systematic review and meta-analysis. Lancet.

[7] Steel Z., Marnane C., Iranpour C., Chey T., Jackson J.W., Patel V., Silove D. (2014). The global prevalence of common mental disorders: A systematic review and meta-analysis 1980–2013. Int. J. Epidemiol..

[8] GBD 2017 Disease and Injury Incidence and Prevalence Collaborators (2018). Global, regional, and national incidence, prevalence, and years lived with disability for 354 diseases and injuries for 195 countries and territories, 1990–2017: A systematic analysis for the Global Burden of Disease Study 2017. Lancet.

[9] Vigo D., Thornicroft G., Atun R. (2016). Estimating the true global burden of mental illness. Lancet Psychiatry.

[10] Tsai W.H., Sung F.C., Chiu L.T., Shih Y.H., Tsai M.C., Wu S.I. (2022). Decreased risk of anxiety in diabetic patients receiving glucagon-like peptide-1 receptor agonist: A nationwide, population-based cohort study. Front. Pharmacol..

[11] Zhao X., Wang M., Wen Z., Lu Z., Cui L., Fu C., Xue H., Liu Y., Zhang Y. (2021). GLP-1 Receptor Agonists: Beyond Their Pancreatic Effects. Front. Endocrinol..

[12] Reich N., Hölscher C. (2022). The neuroprotective effects of glucagon-like peptide 1 in Alzheimer’s and Parkinson’s disease: An in-depth review. Front. Neurosci..

[13] Kim Y.K., Kim O.Y., Song J. (2020). Alleviation of Depression by Glucagon-Like Peptide 1 Through the Regulation of Neuroinflammation, Neurotransmitters, Neurogenesis, and Synaptic Function. Front. Pharmacol..

[14] Mansur R.B., Ahmed J., Cha D.S., Woldeyohannes H.O., Subramaniapillai M., Lovshin J., Lee J.G., Lee J.H., Brietzke E., Reininghaus E.Z. (2017). Liraglutide promotes improvements in objective measures of cognitive dysfunction in individuals with mood disorders: A pilot, open-label study. J. Affect. Disord..

[15] Vadini F., Simeone P.G., Boccatonda A., Guagnano M.T., Liani R., Tripaldi R., Di Castelnuovo A., Cipollone F., Consoli A., Santilli F. (2020). Liraglutide improves memory in obese patients with prediabetes or early type 2 diabetes: A randomized, controlled study. Int. J. Obes..

[16] Anderberg R.H., Richard J.E., Hansson C., Nissbrandt H., Bergquist F., Skibicka K.P. (2016). GLP-1 is both anxiogenic and antidepressant; divergent effects of acute and chronic GLP-1 on emotionality. Psychoneuroendocrinology.

[17] Xie Y., Choi T., Al-Aly Z. (2025). Mapping the effectiveness and risks of GLP1R agonists. Nat. Med..

[18] Thanassoulis G., O’Donnell C.J. (2009). Mendelian randomization: Nature’s randomized trial in the post-genome era. JAMA.

[19] Smith G.D., Ebrahim S. (2003). ’Mendelian randomization’: Can genetic epidemiology contribute to understanding environmental determinants of disease?. Int. J. Epidemiol..

[20] Walker V.M., Davey Smith G., Davies N.M., Martin R.M. (2017). Mendelian randomization: A novel approach for the prediction of adverse drug events and drug repurposing opportunities. Int. J. Epidemiol..

[21] Schmidt A.F., Finan C., Gordillo-Marañón M., Asselbergs F.W., Freitag D.F., Patel R.S., Tyl B., Chopade S., Faraway R., Zwierzyna M. (2020). Genetic drug target validation using Mendelian randomisation. Nat. Commun..

[22] Ference B.A., Ray K.K., Catapano A.L., Ference T.B., Burgess S., Neff D.R., Oliver-Williams C., Wood A.M., Butterworth A.S., Di Angelantonio E. (2019). Mendelian Randomization Study of *ACLY* and Cardiovascular Disease. N. Engl. J. Med..

[23] Sofat R., Hingorani A.D., Smeeth L., Humphries S.E., Talmud P.J., Cooper J., Shah T., Sandhu M.S., Ricketts S.L., Boekholdt S.M. (2010). Separating the mechanism-based and off-target actions of cholesteryl ester transfer protein inhibitors with CETP gene polymorphisms. Circulation.

[24] Hingorani A.D., Kuan V., Finan C., Kruger F.A., Gaulton A., Chopade S., Sofat R., MacAllister R.J., Overington J.P., Hemingway H. (2019). Improving the odds of drug development success through human genomics: Modelling study. Sci. Rep..

[25] Yarmolinsky J., Bouras E., Constantinescu A., Burrows K., Bull C.J., Vincent E.E., Martin R.M., Dimopoulou O., Lewis S.J., Moreno V. (2023). Genetically proxied glucose-lowering drug target perturbation and risk of cancer: A Mendelian randomisation analysis. Diabetologia.

[26] Tang B., Wang Y., Jiang X., Thambisetty M., Ferrucci L., Johnell K., Hägg S. (2022). Genetic Variation in Targets of Antidiabetic Drugs and Alzheimer Disease Risk: A Mendelian Randomization Study. Neurology.

[27] Zhu Y., Li M., Wang H., Yang F., Pang X., Du R., Zhang J., Huang X. (2023). Genetically proxied antidiabetic drugs targets and stroke risk. J. Transl. Med..

[28] Fu K., Si S., Jin X., Zhang Y., Duong V., Cai Q., Li G., Oo W.M., Zheng X., Boer C.G. (2024). Exploring antidiabetic drug targets as potential disease-modifying agents in osteoarthritis. EBioMedicine.

[29] Gold J.M., Goldberg R.W., McNary S.W., Dixon L.B., Lehman A.F. (2002). Cognitive correlates of job tenure among patients with severe mental illness. Am. J. Psychiatry.

[30] Haddad P.M., Correll C.U. (2018). The acute efficacy of antipsychotics in schizophrenia: A review of recent meta-analyses. Ther. Adv. Psychopharmacol..

[31] Siskind D., Hahn M., Correll C.U., Fink-Jensen A., Russell A.W., Bak N., Broberg B.V., Larsen J., Ishøy P.L., Vilsbøll T. (2019). Glucagon-like peptide-1 receptor agonists for antipsychotic-associated cardio-metabolic risk factors: A systematic review and individual participant data meta-analysis. Diabetes Obes. Metab..

[32] Yang Y., Fang H., Xu G., Zhen Y., Zhang Y., Tian J., Zhang D., Zhang G., Xu J. (2018). Liraglutide improves cognitive impairment via the AMPK and PI3K/Akt signaling pathways in type 2 diabetic rats. Mol. Med. Rep..

[33] Iwai T., Suzuki M., Kobayashi K., Mori K., Mogi Y., Oka J.-I. (2009). The influences of juvenile diabetes on memory and hippocampal plasticity in rats: Improving effects of glucagon-like peptide-1. Neurosci. Res..

[34] Palleria C., Leo A., Andreozzi F., Citraro R., Iannone M., Spiga R., Sesti G., Constanti A., De Sarro G., Arturi F. (2017). Liraglutide prevents cognitive decline in a rat model of streptozotocin-induced diabetes independently from its peripheral metabolic effects. Behav. Brain Res..

[35] Chen S., Sun J., Zhao G., Guo A., Chen Y., Fu R., Deng Y. (2017). Liraglutide Improves Water Maze Learning and Memory Performance While Reduces Hyperphosphorylation of Tau and Neurofilaments in APP/PS1/Tau Triple Transgenic Mice. Neurochem. Res..

[36] Long-Smith C.M., Manning S., McClean P.L., Coakley M.F., O’Halloran D.J., Holscher C., O’Neill C. (2013). The diabetes drug liraglutide ameliorates aberrant insulin receptor localisation and signalling in parallel with decreasing both amyloid-β plaque and glial pathology in a mouse model of Alzheimer’s disease. Neuromolecular Med..

[37] Tai J., Liu W., Li Y., Li L., Hölscher C. (2018). Neuroprotective effects of a triple GLP-1/GIP/glucagon receptor agonist in the APP/PS1 transgenic mouse model of Alzheimer’s disease. Brain Res..

[38] Chaves Filho A.M., Cunha N.L., de Souza A.G., Verde-Ramo Soares M., Jucá P.M., de Queiroz T., Souza Oliveira J.V., Valvassori S.S., Barichello T., Quevedo J. (2020). The GLP1R agonist liraglutide reverses mania-like alterations and memory deficits induced by D-amphetamine and augments lithium effects in mice: Relevance for bipolar disorder. Prog. Neuropsychopharmacol. Biol. Psychiatry.

[39] Kutlu M.D., Kose S., Akillioglu K. (2023). GLP-1 agonist Liraglutide prevents MK-801-induced schizophrenia-like behaviors and BDNF, CREB, p-CREB, Trk-B expressions in the hippocampus and prefrontal cortex in Balb/c mice. Behav. Brain Res..

[40] Flintoff J., Kesby J.P., Siskind D., Burne T.H. (2021). Treating cognitive impairment in schizophrenia with GLP1RA: An overview of their therapeutic potential. Expert. Opin. Investig. Drugs.

[41] Yu Y., Fu Y., Yu Y., Tang M., Sun Y., Wang Y., Zhang K., Li H., Guo H., Wang B. (2023). Investigating the shared genetic architecture between schizophrenia and body mass index. Mol. Psychiatry.

[42] Rødevand L., Rahman Z., Hindley G.L., Smeland O.B., Frei O., Tekin T.F., Kutrolli G., Bahrami S., Hoseth E.Z., Shadrin A. (2023). Characterizing the Shared Genetic Underpinnings of Schizophrenia and Cardiovascular Disease Risk Factors. Am. J. Psychiatry.

[43] Aoki R., Saito T., Ninomiya K., Shimasaki A., Ashizawa T., Ito K., Ikeda M., Iwata N. (2022). Shared genetic components between metabolic syndrome and schizophrenia: Genetic correlation using multipopulation data sets. Psychiatry Clin. Neurosci..

[44] Yaribeygi H., Rashidy-Pour A., Atkin S.L., Jamialahmadi T., Sahebkar A. (2021). GLP-1 mimetics and cognition. Life Sci..

[45] Bora E., Akdede B.B., Alptekin K. (2017). The relationship between cognitive impairment in schizophrenia and metabolic syndrome: A systematic review and meta-analysis. Psychol. Med..

[46] Bocarsly M.E., Fasolino M., Kane G.A., LaMarca E.A., Kirschen G.W., Karatsoreos I.N., McEwen B.S., Gould E. (2015). Obesity diminishes synaptic markers, alters microglial morphology, and impairs cognitive function. Proc. Natl. Acad. Sci. USA.

[47] Fornari Laurindo L., Barbalho S.M., Guiguer E.L., Soares de Souza M.S., de Souza G.A., Fidalgo T.M., Araújo A.C., de Souza Gonzaga H.F., de Bortoli Teixeira D., de Oliveira Silva Ullmann T. (2022). GLP-1a: Going beyond Traditional Use. Int. J. Mol. Sci..

[48] Detka J., Głombik K. (2021). Insights into a possible role of glucagon-like peptide-1 receptor agonists in the treatment of depression. Pharmacol. Rep..

[49] Chen X., Zhao P., Wang W., Guo L., Pan Q. (2024). The Antidepressant Effects of GLP1R Agonists: A Systematic Review and Meta-Analysis. Am. J. Geriatr. Psychiatry.

[50] Li J.R., Cao J., Wei J., Geng W. (2023). Case Report: Semaglutide-associated depression: A report of two cases. Front. Psychiatry.

[51] Selman A., Dai J., Driskill J., Reddy A.P., Reddy P.H. (2025). Depression and obesity: Focus on factors and mechanistic links. Biochim. Biophys. Acta Mol. Basis Dis..

[52] Tobaiqy M., Elkout H. (2024). Psychiatric adverse events associated with semaglutide, liraglutide and tirzepatide: A pharmacovigilance analysis of individual case safety reports submitted to the EudraVigilance database. Int. J. Clin. Pharm..

[53] McElroy S.L., Mori N., Guerdjikova A.I., Keck P.E. (2018). Would glucagon-like peptide-1 receptor agonists have efficacy in binge eating disorder and bulimia nervosa? A review of the current literature. Med. Hypotheses.

[54] Sun D., Gao W., Hu H., Zhou S. (2022). Why 90% of clinical drug development fails and how to improve it?. Acta Pharm. Sin. B.

[55] Gill D., Georgakis M.K., Walker V.M., Schmidt A.F., Gkatzionis A., Freitag D.F., Finan C., Hingorani A.D., Howson J.M., Burgess S. (2021). Mendelian randomization for studying the effects of perturbing drug targets. Wellcome Open Res..

[56] Burgess S., Butterworth A., Malarstig A., Thompson S.G. (2012). Use of Mendelian randomisation to assess potential benefit of clinical intervention. BMJ.

[57] Võsa U., Claringbould A., Westra H.J., Bonder M.J., Deelen P., Zeng B., Kirsten H., Saha A., Kreuzhuber R., Yazar S. (2021). Large-scale cis- and trans-eQTL analyses identify thousands of genetic loci and polygenic scores that regulate blood gene expression. Nat. Genet..

[58] Mahajan A., Spracklen C.N., Zhang W., Ng M.Y., Petty L.E., Kitajima H., Yu G.Z., Rüeger S., Speidel L., Kim Y.J. (2022). Multi-ancestry genetic study of type 2 diabetes highlights the power of diverse populations for discovery and translation. Nat. Genet..

[59] Yengo L., Sidorenko J., Kemper K.E., Zheng Z., Wood A.R., Weedon M.N., Frayling T.M., Hirschhorn J., Yang J., Visscher P.M. (2018). Meta-analysis of genome-wide association studies for height and body mass index in ∼700000 individuals of European ancestry. Hum. Mol. Genet..

[60] Demontis D., Walters G.B., Athanasiadis G., Walters R., Therrien K., Nielsen T.T., Farajzadeh L., Voloudakis G., Bendl J., Zeng B. (2023). Genome-wide analyses of ADHD identify 27 risk loci, refine the genetic architecture and implicate several cognitive domains. Nat. Genet..

[61] Kurki M.I., Karjalainen J., Palta P., Sipilä T.P., Kristiansson K., Donner K.M., Reeve M.P., Laivuori H., Aavikko M., Kaunisto M.A. (2023). FinnGen provides genetic insights from a well-phenotyped isolated population. Nature.

[62] Watson H.J., Yilmaz Z., Thornton L.M., Hübel C., Coleman J.I., Gaspar H.A., Bryois J., Hinney A., Leppä V.M., Mattheisen M. (2019). Genome-wide association study identifies eight risk loci and implicates metabo-psychiatric origins for anorexia nervosa. Nat. Genet..

[63] Otowa T., Hek K., Lee M., Byrne E.M., Mirza S.S., Nivard M.G., Bigdeli T., Aggen S.H., Adkins D., Wolen A. (2016). Meta-analysis of genome-wide association studies of anxiety disorders. Mol. Psychiatry.

[64] Grove J., Ripke S., Als T.D., Mattheisen M., Walters R.K., Won H., Pallesen J., Agerbo E., Andreassen O.A., Anney R. (2019). Identification of common genetic risk variants for autism spectrum disorder. Nat. Genet..

[65] Mullins N., Forstner A.J., O’Connell K.S., Coombes B., Coleman J.I., Qiao Z., Als T.D., Bigdeli T.B., Børte S., Bryois J. (2021). Genome-wide association study of more than 40,000 bipolar disorder cases provides new insights into the underlying biology. Nat. Genet..

[66] Howard D.M., Adams M.J., Clarke T.-K., Hafferty J.D., Gibson J., Shirali M., Coleman J.I., Hagenaars S.P., Ward J., Wigmore E.M. (2019). Genome-wide meta-analysis of depression identifies 102 independent variants and highlights the importance of the prefrontal brain regions. Nat. Neurosci..

[67] Nievergelt C.M., Maihofer A.X., Klengel T., Atkinson E.G., Chen C.-Y., Choi K.W., Coleman J.R.I., Dalvie S., Duncan L.E., Gelernter J. (2019). International meta-analysis of PTSD genome-wide association studies identifies sex- and ancestry-specific genetic risk loci. Nat. Commun..

[68] Schizophrenia Working Group of the Psychiatric Genomics Consortium (2014). Biological insights from 108 schizophrenia-associated genetic loci. Nature.

[69] Johnson E.C., Demontis D., Thorgeirsson T.E., Walters R.K., Polimanti R., Hatoum A.S., Sanchez-Roige S., Paul S.E., Wendt F.R., Clarke T.-K. (2020). A large-scale genome-wide association study meta-analysis of cannabis use disorder. Lancet Psychiatry.

[70] Sanchez-Roige S., Palmer A.A., Fontanillas P., Elson S.L., Adams M.J., Howard D.M., Edenberg H.J., Davies G., 23andMe Research Team, Substance Use Disorder Working Group of the Psychiatric Genomics Consortium (2019). Genome-Wide Association Study Meta-Analysis of the Alcohol Use Disorders Identification Test (AUDIT) in Two Population-Based Cohorts. Am. J. Psychiatry.

[71] Wishart D.S., Feunang Y.D., Guo A.C., Lo E.J., Marcu A., Grant J.R., Sajed T., Johnson D., Li C., Sayeeda Z. (2018). DrugBank 5.0: A major update to the DrugBank database for 2018. Nucleic Acids Res..

[72] Gaulton A., Hersey A., Nowotka M., Bento A.P., Chambers J., Mendez D., Mutowo P., Atkinson F., Bellis L.J., Cibrián-Uhalte E. (2017). The ChEMBL database in 2017. Nucleic Acids Res..

[73] Rosoff D.B., Bell A.S., Jung J., Wagner J., Mavromatis L.A., Lohoff F.W. (2022). Mendelian Randomization Study of PCSK9 and HMG-CoA Reductase Inhibition and Cognitive Function. J. Am. Coll. Cardiol..

[74] Yan R., Liu L., Tzoulaki I., Fan J., Targher G., Yuan Z., Zhao J. (2024). Genetic Evidence for GLP-1 and GIP Receptors as Targets for Treatment and Prevention of MASLD/MASH. Liver Int..

[75] Lawlor D.A., Harbord R.M., Sterne J.A., Timpson N., Davey Smith G. (2008). Mendelian randomization: Using genes as instruments for making causal inferences in epidemiology. Stat. Med..

[76] Drucker D.J., Holst J.J. (2023). The expanding incretin universe: From basic biology to clinical translation. Diabetologia.

[77] Burgess S., Davey Smith G., Davies N.M., Dudbridge F., Gill D., Glymour M.M., Hartwig F.P., Kutalik Z., Holmes M.V., Minelli C. (2023). Guidelines for performing Mendelian randomization investigations: Update for summer 2023. Wellcome Open Res..

[78] Burgess S., Butterworth A., Thompson S.G. (2013). Mendelian randomization analysis with multiple genetic variants using summarized data. Genet. Epidemiol..

[79] Hartwig F.P., Davey Smith G., Bowden J. (2017). Robust inference in summary data Mendelian randomization via the zero modal pleiotropy assumption. Int. J. Epidemiol..

[80] Bowden J., Davey Smith G., Burgess S. (2015). Mendelian randomization with invalid instruments: Effect estimation and bias detection through Egger regression. Int. J. Epidemiol..

[81] Bowden J., Davey Smith G., Haycock P.C., Burgess S. (2016). Consistent Estimation in Mendelian Randomization with Some Invalid Instruments Using a Weighted Median Estimator. Genet. Epidemiol..

[82] Bowden J., Del Greco M.F., Minelli C., Zhao Q., Lawlor D.A., Sheehan N.A., Thompson J., Davey Smith G. (2019). Improving the accuracy of two-sample summary-data Mendelian randomization: Moving beyond the NOME assumption. Int. J. Epidemiol..

[83] Carter A.R., Sanderson E., Hammerton G., Richmond R.C., Davey Smith G., Heron J., Taylor A.E., Davies N.M., Howe L.D. (2021). Mendelian randomisation for mediation analysis: Current methods and challenges for implementation. Eur. J. Epidemiol..

[84] Xu L., Borges M.C., Hemani G., Lawlor D.A. (2017). The role of glycaemic and lipid risk factors in mediating the effect of BMI on coronary heart disease: A two-step, two-sample Mendelian randomisation study. Diabetologia.

[85] Zhang J., Chen Z., Pärna K., van Zon S.R., Snieder H., Thio C.L. (2022). Mediators of the association between educational attainment and type 2 diabetes mellitus: A two-step multivariable Mendelian randomisation study. Diabetologia.

[86] Zhao X., Lynch J.G., Chen Q. (2010). Reconsidering Baron and Kenny: Myths and Truths about Mediation Analysis. J. Consum. Res..

